# Unveiling the Effects of Processing Parameters on Microstructure, Mechanical Properties, and Corrosion Resistance of High-Nb TiAl Alloy Fabricated by Laser Powder Bed Fusion

**DOI:** 10.3390/ma19071328

**Published:** 2026-03-27

**Authors:** Gaoxi Wang, Ziwen Xie, Dongxu Zhang, Chenglong Ma

**Affiliations:** 1 Ulster College, Shaanxi University of Science and Technology, Xi’an 710021, China; 202315020203@sust.edu.cn; 2 Jiangsu Key Laboratory of Advanced Food Manufacturing Equipment & Technology, School of Mechanical Engineering, Jiangnan University, Wuxi 214122, China; 7230809016@stu.jiangnan.edu.cn; 3 College of Mechanical and Electrical Engineering, Shaanxi University of Science and Technology, Xi’an 710021, China; 4 Jiangsu Province Engineering Research Center of Micro-Nano Additive and Subtractive Manufacturing, Wuxi 214122, China

**Keywords:** laser powder bed fusion, high-Nb TiAl alloy, laser energy density, wear resistance, corrosion resistance

## Abstract

**Highlights:**

**What are the main findings?**

- Implementing an optimized VED facilitated near-full densification with a substantial mitigation of internal porosity and micro-cracks.
- Driven by the non-equilibrium solidification, the microstructure was primarily constituted by γ, α2, and β/B2 phases.
- Specimens processed under optimized conditions demonstrated a minimum friction coefficient and corrosion current density accompanied by enhanced wear and corrosion resistance, respectively.

**What are the implications of the main findings?**

- Accurate regulation of the VED is essential for suppressing defect formation such as porosity and cracking in high-Nb TiAl alloys.
- Optimized high-Nb TiAl alloys exhibit promising applicability under severe worn and corrosive environments.
- These findings systematically elucidate the correlation between thermal input, phaser evolution, and material performance.

**Abstract:**

This study elucidates the impact of laser volumetric energy density (VED) on the densification behavior, microstructural evolution, wear resistance, and corrosion resistance of high-Nb TiAl alloys fabricated via laser powder bed fusion (LPBF). Experimental characterization results showed that relative density first increased and then decreased with increasing VED, reaching a maximum density of 97.13% at 66.67 J/mm^3^. Across the process windows, the high-Nb TiAl alloys were primarily composed of γ-TiAl, α2-Ti3Al, and β/B2 phases with varied proportions. Mechanical property analysis showed that the alloy attained a maximum average hardness of 422 HV_0.5_ at 81.48 J/mm^3^, due to the accumulation of harder α2 and B2 phases. However, the high-Nb TiAl alloys fabricated at 66.67 J/mm^3^ exhibited excellent wear resistance, as evidenced by wear track widths and depths of 971.71 μm and 21.83 μm, respectively. Abrasive and oxidative wear were identified as the primary mechanisms. Meanwhile, this specimen also exhibited excellent corrosion resistance, a corrosion current density of 1.421 × 10^−6^ A/cm^2^, attributed to the coupled dense passive film of TiO_2_ and Al_2_O_3_ that prevented chloride ingress. The findings in this work may provide a critical experimental reference and theoretical underpinnings for LPBF-fabricated lightweight structural materials.

## 1. Introduction

Driven by the escalating imperative for lightweight structural components capable of withstanding extreme thermal environments, high-niobium titanium aluminum (high-Nb TiAl) alloys have emerged as a leading candidate for next-generation aerospace, automotive propulsion, and power generation applications. The strategic importance of this alloy stems from its exceptional synergy of low mass density and high specific strength, coupled with superior oxidation resistance and mechanical integrity at elevated temperatures [1,2]. Nevertheless, under aggressive service conditions, wear and corrosion generally dictate failure mechanisms and severely compromise the engineering reliability and longevity of these alloys [3]. Furthermore, the intrinsic brittleness and restricted fracture toughness of the material present formidable obstacles to conventional thermomechanical processing, creating a critical barrier to the fabrication of complex geometries required for widespread industrial adoption [4].

Additive Manufacturing (AM) has emerged as a transformative near-net-shape processing strategy to circumvent conventional manufacturing constraints [5]. By enabling the direct synthesis of fully dense metallic components with intricate geometries and refined microstructures, this technology establishes a novel paradigm for the integral manufacturing of high-Nb TiAl alloys. Prominent methodologies within the metal AM landscape include Electron Beam Powder Bed Fusion (EPBF), Laser Powder Bed Fusion (LPBF), and Laser Metal Deposition (LMD) [6,7,8]. Among these technological approaches, LPBF is distinguished by the deployment of high-energy laser irradiation to induce selective melting and rapid solidification in a layer-by-layer manner. The exceptional dimensional precision and unprecedented geometric flexibility derived from this specific thermal history render LPBF a preeminent processing route for high-performance structural materials [9]. However, investigations indicate that the rapid solidification kinetics and steep spatial thermal gradients inherent to LPBF processes predispose high-Nb TiAl alloys to extensive cracking and porosity by driving defect nucleation and propagation throughout solidification and subsequent cooling [10]. Consequently, the primary countermeasure adopted preheats the substrate to elevated temperatures. Exemplifying this efficacy, experimental studies by Gussone and Kazuhiro demonstrated that maintaining substrate temperatures exceeding the brittle-ductile transition temperature facilitates the fabrication of TiAl specimens exhibiting negligible cracking susceptibility [11,12]. Nevertheless, reliance on such high-temperature requirements necessitates sophisticated heat-resistant hardware and precipitates rapid oxidation of the powder feedstock. These limitations entail prohibitive capital and operational expenditures that severely impede widespread industrial adoption. Consequently, the synergistic regulation of defect suppression and microstructural evolution through precise parameter control constitutes a promising avenue for advancing LPBF-fabricated high-Nb TiAl alloys.

Within the multidimensional parameter windows of LPBF, processing variables such as laser power, scanning velocity, hatch spacing, and layer thickness orchestrate the stability of the manufacturing process. By modulating melt pool dynamics and thermal cycling, these parameters fundamentally govern the solidification kinetics, thereby determining the resultant densification behavior, phase constitution, and microstructural morphology [13,14,15]. Specifically, Li et al. [16] elucidated the correlation between energy input parameters, specifically laser power, scanning velocity, and the densification behavior of LPBF-fabricated Ti-45Al-8Nb, achieving a relative density of 98.9% via parameter tailoring. Wang et al. [17] highlighted the critical role of hatch spacing in the fabricability of Ti-48Al-2Cr-8Nb. Their approach successfully mitigated solidification cracking and yielded a near-α single-phase structure with a relative density reaching 99.23%. Park et al. [18] attributed these benefits to an intrinsic heat-treatment effect driven by narrow hatch spacing, characterized by sufficient remelting and reduced cooling rates, which effectively suppresses crack initiation while promoting complex phase transformations. Furthermore, Li et al. [19] examined the effects of scanning velocity on Ti-45Al-2Cr-5Nb, demonstrating that optimized processing strategies not only ensure structural integrity but also impart nano-hardness and compressive properties superior to those of conventionally processed counterparts. Although significant scholarly attention has been devoted to crack initiation mechanisms, defect mitigation, and microstructural evolution in LPBF-fabricated high-Nb TiAl alloys, a comprehensive understanding regarding the synergistic impact of volumetric energy density (VED) on performance remains elusive. Given the prospective deployment of these alloys in severe tribological and corrosive environments [20,21], it is imperative to elucidate the intrinsic correlations linking process-induced variations, such as microstructural and defect characteristics, to their resulting wear and corrosion resistance. Nevertheless, systematic investigations into the friction and electrochemical behavior of LPBF high-Nb TiAl remain scarce. Specifically, the field lacks a rigorous analysis of the process-structure-property mapping that connects laser energy inputs to the interplay among microstructural characteristics, tribological performance, and corrosion behavior.

Building upon this background, the present study elucidates the impact of VED, acting as a pivotal processing variable, on the microstructural, mechanical performance, and electrochemical response of high-Nb TiAl alloys fabricated via LPBF. By employing a systematic characterization strategy encompassing densification behavior, phase constituent evolution, microstructural morphology, frictional behavior assessment, and potentiodynamic analysis, this work delineates the underlying structure-property relationships governed by variations in thermal input. Ultimately, these findings establish a theoretical framework and empirical guidelines for parameter optimization, facilitating the fabrication of high-integrity high-Nb TiAl components suitable for industrial deployment.

## 2. Materials and Methods

### 2.1. Specimen Preparation

**Figure 1:**
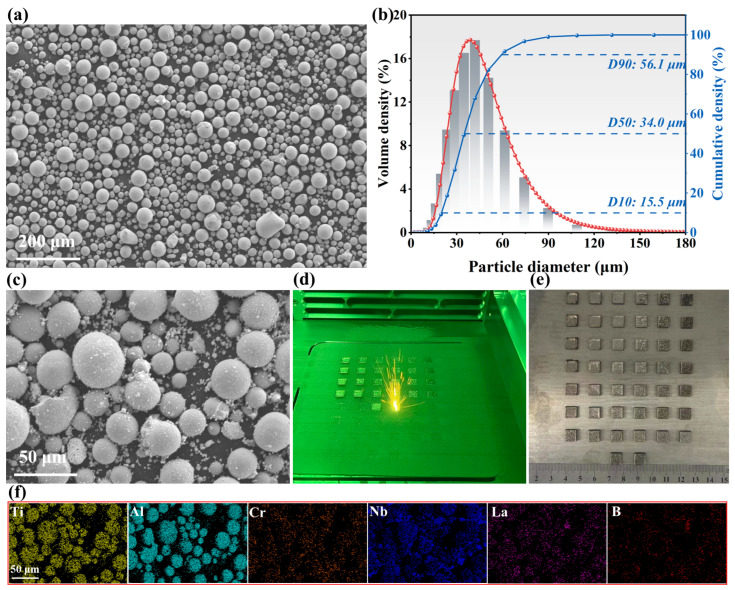
(**a**) TiAl powder; (**b**) Particle size distribution curve for TiAl powder; (**c**) The high-Nb TiAl mixture powder; (**d**) Actual LPBF process; (**e**) As-fabricated specimens; (**f**) EDS analysis of composite powder particles.

The TiAl-based composite was fabricated using an iSLM 160 LPBF system (ZRapid Tech, Suzhou, China) equipped with a continuous 1064 nm Yb-YAG fiber laser and a 100 μm laser spot size. A Ti6Al4V plate with dimensions of 160 × 160 × 35 mm^3^ served as the build substrate. The Ti48Al2Cr2Nb powders were prepared by gas atomization with a particle size distribution of 15~53 μm, as shown in Figure 1. Specifically, Figure 1a,b illustrate the powder morphology and the particle size distribution histogram, respectively. To enhance the processability and physicochemical performance of the LPBF-fabricated TiAl alloy, Nb nanoparticles and sub-micron LaB_6_ particles were introduced into the matrix feedstock. Nb nanoparticles were incorporated to achieve the target high-Nb composition and promote thermodynamic β-stabilization, whereas sub-micron LaB_6_ was introduced to mitigate the intrinsic cracking susceptibility of TiAl by LPBF [22]. The mixture powders were mechanically ball-milled to ensure homogeneity. The ball milling was conducted at 200 r/min with a ball-to-powder ratio of 2:1 for 2 h, including a 5 min cooling intervals every 15 min. The morphology of the mixture composite powder is visualized in Figure 1c. Prior to fabrication, the powders were dried at 105 °C for a minimum of 8 h. Table 1 details the critical processing parameters, including laser power (*P*), scanning speed (*v*), hatch spacing (*h*), and layer thickness (*t*). The laser volumetric energy density (*VED*) can be calculated using the following formula:

(1) $$VED=\frac{P}{vht}$$

The bi-directional scanning strategy with a 67° interlayer rotation vector was employed to fabricate cubic specimens measuring 10 × 10 × 10 mm^3^. In addition, a fixed scanning interval of 50 μm and a powder bed layer thickness of 30 μm were maintained. Owing to the high oxygen affinity of the titanium melt at elevated temperatures, the forming process was carried out under a controlled atmosphere maintaining oxygen concentration below 100 ppm.

### 2.2. Microstructural Characterization

Specimens for microstructural characterization were extracted from the substrate via wire electrical discharge machining. The plane parallel to the building direction was selected for observation. To assess physical integrity, the relative density measurements were conducted employing Archimedes’ principle. Standard metallographic preparation involved grinding with silicon carbide papers with 400 to 2500 grit, followed by precision polishing to a mirror-like finish with a diamond suspension. To reveal microstructural features, the polished specimens’ surfaces were etched with Kroll’s reagent (HF: HNO_3_: H_2_O = 1:3:46) for 10 to 15 s. The microstructural morphology of high-Nb TiAl materials was characterized using a scanning electron microscope (SEM; Zeiss Gemini Sigma 300, Oberkochen, Germany) operated at an accelerating voltage of 15 kV. Elemental distribution was concurrently mapped via an integrated energy-dispersive X-ray spectroscopy (EDS) system. Phase constituent analysis was executed on the Bruker D8 Advance X-ray diffractometer (XRD) with Cu-Kα radiation. Diffraction patterns were acquired with a step size of 0.02° and a dwell time of 0.2 s/step. Subsequent phase identification was processed using Jade 6 software.

### 2.3. Mechanical Properties and Corrosion Behavior

The hardness values of TiAl-based materials were measured using an HVS-1000ZCM-XY tester (Shanghai Suoyan Testing Instrument Co., Ltd., Shanghai, China) at a load of 500 gf and a dwell period of 10 s with five repeated measurements taken at different positions. Tribological properties were subsequently evaluated on the MFT-5000 tribometer (Rtec Co., San Jose, CA, USA) with a normal load of 30 N, a frequency of 1 Hz, a sliding stroke of 5 mm, and a test duration of 30 min. A Si_3_N_4_ sphere with a 6 mm diameter served as the abrasive surface. Post-wear topographical analysis was performed using an MFD-D (Retc Co., San Jose, CA, USA) white light interferometer to obtain the 3D morphology of the worn surfaces. The specific wear rate in this work was calculated by the following formula [23,24]:

(2) $$V=B\left(\frac{\pi R^{2}}{180}\arcsin\frac{b}{2R}-\frac{b}{2}\sqrt{R^{2}-\frac{b^{2}}{4}}\right)$$

(3) $$L=2\pi Rv_{0}t$$

(4) $$W=\frac{V}{L\times N}$$

where *V* is the wear volume, *B* is the wear length, *R* is the radius of the Si_3_N_4_ ball, *b* is the wear width, *L* is the sliding distance, *v*_0_ is the sliding speed, *W* is the wear rate, and *N* is the applied force. All measurements were repeated three times under identical conditions to ensure reproducibility and statistical reliability.

Electrochemical corrosion behavior was investigated in 3.5 wt.% NaCl solution using a CHI760e electrochemical workstation (CH Instruments, Inc. Austin, TX, USA) with a three-electrode system consisting of the LPBF-fabricated specimens as a working electrode, a 15 × 15 mm^2^ platinum thin sheet as a counter electrode, and a saturated calomel electrode (AgCl) as a reference electrode. To ensure the thermodynamic stability of the passive film, the open-circuit potential (OCP) was monitored for 6000 s before further testing. Electrochemical impedance spectroscopy (EIS) measurements were conducted at the OCP using a sinusoidal perturbation amplitude of 0.005 V over a frequency range from 10^5^ Hz to 10^−2^ Hz. The obtained spectral data were fitted to equivalent electrical circuits using Zview 3.6 software. Potentiodynamic polarization curves were recorded at a scan rate of 2 mV/s across a potential window from −1.0 V to 1.0 V. All electrochemical measurements were performed using a minimum of three replicate samples to verify the reproducibility of the results. The reported data represent the mean values, with error margins given as the standard deviation.

## 3. Results and Discussion

### 3.1. Densification and Defects

**Figure 2:**
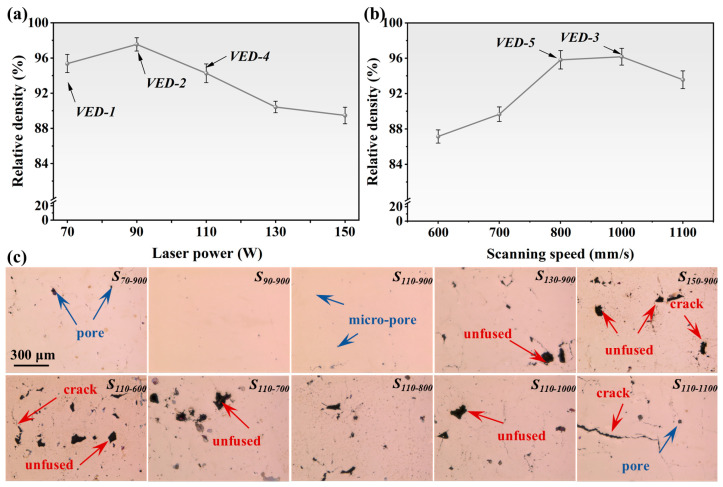
The relative density and defects of specimens fabricated by LPBF under varying volume energy densities: (**a**) Various laser powers; (**b**) Various scanning speeds; (**c**) Metallographic morphology of specimens.

Optimization of LPBF processing parameters is a prerequisite for achieving dense, crack-free fabrication of high-Nb TiAl alloys. The influence of the primary process parameter has been preliminarily investigated in previous studies [25,26]. Figure 2 illustrates the impacts of laser power (*P*) and scanning speed (*v*) on the relative density and defect characteristics of the processed specimens. At a constant *v*, the relative density exhibited a non-monotonic response to *P*, initially increasing and subsequently decreasing with increasing *P*. Under low-*P* conditions, insufficient VED resulted in a smaller molten pool size and abbreviated solidification time, which restricted melt spreading and metallurgical bonding. As a consequence, extensive lack-of-fusion porosity and interconnected cracking were observed. Elevating the *P* facilitated defect remediation by stabilizing the melt pool dynamics and enhancing the liquid wettability. Notably, exceeding the appropriate *P* value precipitated a pronounced deterioration in densification. The recurrence of porosity at high VED was associated with keyhole-induced instabilities, triggered by the Al evaporation under excessive laser irradiation. The violent vapor recoil pressure destabilized the melt pool, leading to keyhole collapse, gas entrainment, and porosity formation [27]. Analogous trends characterize the response of relative density to *v* variations at constant *P* were observed in Figure 2b. In the low-*v* regime, prolonged thermal exposure intensified the convection and evaporation of the molten pool. Although this phenomenon was conducive to powder fusion, high VED induced severe spattering and ejection, thus compromising molten pool stability and forming quality. Modulating the *v* to intermediate levels could harmonize the energy input and ensure complete melting, which are prerequisites for achieving superior densification. When the scanning speed was further increased, insufficient interaction time between the laser and the powder resulted in inadequate thermal energy for fusion. This energy deficit led to the retention of unfused powder particles, which impaired metallurgical bonding and generated lack-of-fusion defects. The presence of discontinuity regions reduced the powder bed density and disrupted subsequent deposition, culminating in severe defect propagation [28]. Governed by the above parametric analysis, five specimens with superior relative densities were selected for further comprehensive characterization. To systematically elucidate the effects of VED on microstructural evolution, mechanical properties, and corrosion behavior, these specimens were designated VED-1(*S*_70-900_), VED-2(*S*_90-900_), VED-3(*S*_110-1000_), VED-4(*S*_110-900_), and VED-5(*S*_110-800_), respectively. Briefly, at a VED of 66.67 J/mm^3^, the relative density of S2 was 97.55%, while the relative density of S6 was only 87.14%.

### 3.2. Microstructure Characterization

**Figure 3:**
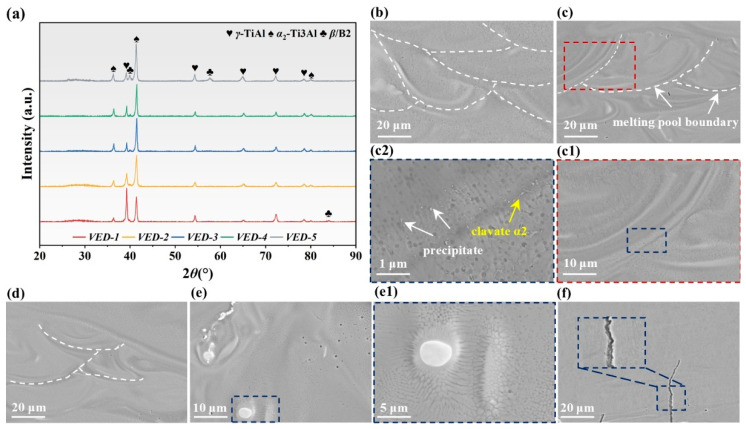
(**a**) XRD diffraction patterns; the microstructure of high-Nb TiAl alloys: (**b**) VED-1; (**c**) VED-2; (**c1**) Enlarged view of (**c**); (**c2**) Enlarged view of (**c1**); (**d**) VED-3; (**e**) VED-4; (**e1**) Enlarged view of (**e**); (**f**) VED-5.

Figure 3a depicts the XRD patterns of the high-Nb TiAl alloys fabricated by LPBF under various VEDs. It was found that γ-TiAl, α2-Ti3Al, and β/B2 were the constituent phases in all specimens. At a relatively low VED, the specimen exhibited pronounced diffraction peaks corresponding to the coexistence of γ and α2 phases, implying that the phase constitution was dominated by intermetallics. With increasing VED, the elevated melt pool temperature and localized energy input significantly altered the solidification dynamics, although the elevated temperatures likely intensified the localized vaporization of the low-boiling-point Al element, which shifts the local composition toward the Ti-rich region, suppressing the formation of the Al-rich γ-TiAl phase and promoting the stabilization of the Ti-rich α2-Ti3Al phase. More importantly, the extremely high cooling rates inherent to the LPBF process impose a strongly non-equilibrium solidification pathway. Rapid quenching drastically reduced the time available for the sluggish diffusional solid-state transformation α → γ, thereby kinetically suppressing the widespread formation of fully ordered γ-TiAl lamellae. Furthermore, as a strong β-stabilizing element in TiAl-based alloy, Nb significantly broadened the β phase region. During the non-equilibrium rapid solidification process, the presence of Nb hindered the diffusional β → α transformation, facilitating the retention of the high-temperature β phase at room temperature in the form of the ordered B2 structure [29]. As a result, an α2 + β/B2 phase microstructure is preferentially established in specimens processed at high VED.

Microstructural characterization of high-Nb TiAl alloys at different VEDs was performed, as illustrated in Figure 3b–d. The specimens displayed a characteristic hierarchy of overlapping stacked molten pools, delineated by arc-shaped boundaries or fish-scale morphologies. Influenced by the heat-flow direction, the solidification substructure comprised epitaxially grown columnar grains extending towards the melt pool center. As the solidification front advances, the decrease in temperature gradient (*G*) coupled with the increase in solidification rate (*R*) reduces the *G*/*R* ratio, promoting the transformation from columnar to equiaxed (CET). Therefore, the interior of the melt pool was dominated by fine equiaxed grains. It was noteworthy that a large amount of needle-like and short rod-like α2 phases were precipitated, as shown in Figure 3(c2). These features were primarily attributed to the eutectoid transformation α → α2 + γ, the ordering transformation α → α2, and the non-diffusive martensitic transformation α → β during the rapid melting and solidification process of LPBF [30]. Concurrently, significant Nb segregation within the melt pool was observed because the solute diffusion was limited by rapid cooling. This enrichment stabilized the β phase, which remained as ordered B2. Notably, this interfacial Nb segregation disrupted epitaxial growth, effectively promoting grain refinement, as shown in Figure 3(e1). This unique behavior could mitigate crack initiation and intergranular propagation. However, distinct intergranular microcracks traversing the melt pool are evident in Figure 3f, attributed to the intrinsic brittleness of TiAl intermetallics, compounded by the severe thermal gradients and residual stresses inherent to the LPBF process at a high VED value [31].

**Figure 4:**
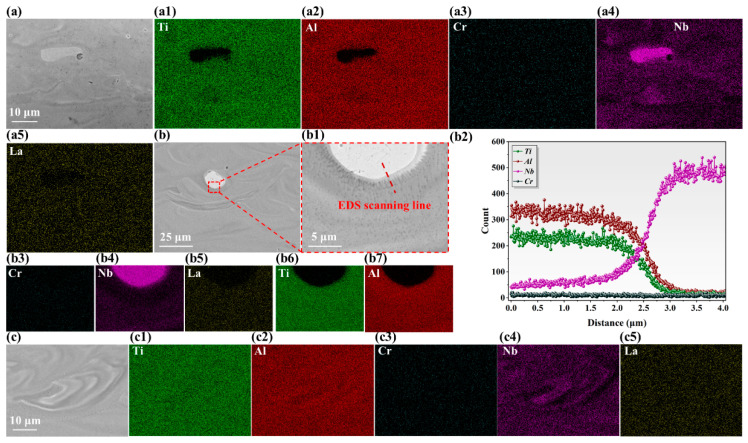
Element mapping analysis of high-Nb TiAl alloys: (**a**) VED-1; (**a1**–**a5**) EDS scanning results shown in (**a**); (**b**) VED-2; (**b1**) Enlarged view of (**b**); (**b2**) the EDS line scanning result in the (**b1**); (**b3**–**b7**) EDS scanning results shown in (**b**); (**c**) VED-4; (**c1**–**c5**) EDS scanning results shown in (**c**).

As presented in Figure 4, the EDS analysis results corroborated the segregation phenomenon of the Nb element within the melt pool. It was noted that although increasing the energy input theoretically promoted solute homogenization by enhanced diffusion, the propensity of cracking paradoxically increased due to intensified thermal stresses. However, the segregation observed at low VED was not entirely detrimental. As shown in Figure 4(b2), the Nb-enriched regions maintained a coherent interface with the TiAl matrix. Furthermore, Nb enrichment not only significantly hindered epitaxial columnar growth by promoting heterogeneous nucleation but also effectively impeded transgranular crack propagation.

### 3.3. Mechanical Properties

**Figure 5:**
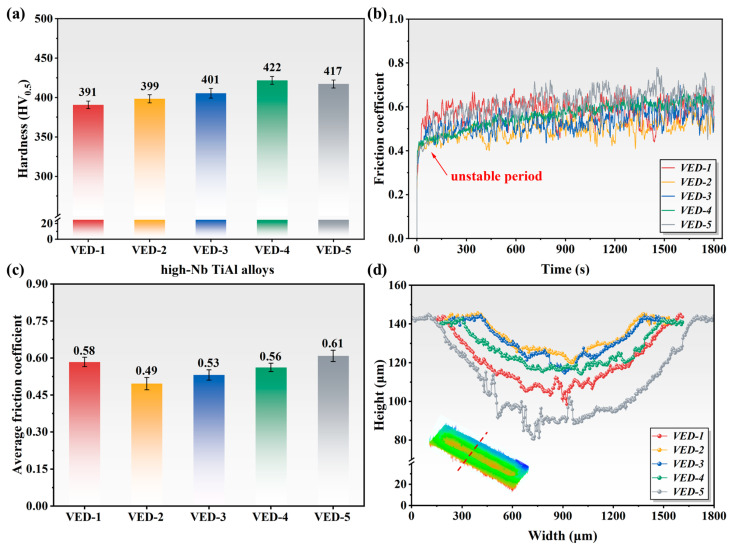
Mechanical properties of high-Nb TiAl alloys: (**a**) Hardness; (**b**) Friction. coefficient curves; (**c**) Average friction wear coefficient; (**d**) Wear section curves.

Figure 5 illustrates the hardness and tribological performance of the high-Nb TiAl specimens fabricated at different VEDs. The marginal upward trend in hardness was observed with increasing energy density, peaking at 421 ± 5 HV_0.5_. This influence was primarily ascribed to the increased volume fraction of the harder α2 and B2 phases relative to the softer γ matrix under high energy input. The coefficient of friction (COF) curves delineated the wear evolution into the run-in period and stable wear stage. During the initial wear stage, the contact interface was dominated by asperities, resulting in localized point contact. The high shear stresses generated induced significant fluctuation in the COF. As sliding progresses into the stable wear regime, the COF oscillation amplitude increases. This phenomenon was attributed to the generation, comminution, and oxidation of wear debris. Specifically, the average COF values for VED-1 to VED-5 were 0.58, 0.49, 0.53, 0.56, and 0.61, respectively. It was noted that the VED-2 specimen exhibited the lowest COF, implying superior tribological stability. Conversely, although the VED-5 specimen possesses higher hardness, it manifested a significantly higher COF. This anomaly arose because internal defects, such as pores and cracks, in high-Nb TiAl alloys acted as stress concentrators and sources of debris, accelerating abrasive wear. Notably, the counterintuitive inverse correlation between hardness and wear resistance in VED-5 acts as a function of its microstructural heterogeneity. While the higher volume fraction of the ordered β/B2 phase contributes to superior hardness, it induces a significant elastic-plastic mismatch with the surrounding ductile matrix. Subjected to tribological loading, the intrinsic paucity of independent slip systems within the lattice necessitates rapid dislocation pile-up at the semi-coherent matrix interfaces. Such localized stress concentrations precipitate interfacial debonding and crack nucleation rather than plastic accommodation. Consequently, the hard precipitates undergo brittle spallation, transitioning the wear mechanism to severe three-body abrasion.

**Figure 6:**
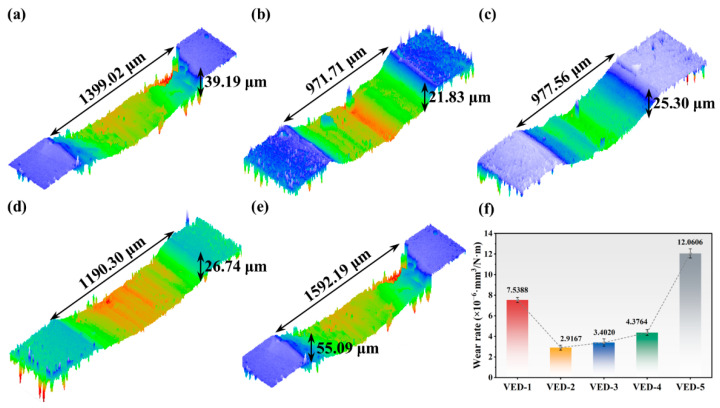
(**a**–**e**) Surface friction 3D morphology of the VED-1 to VED-5 specimens; (**f**) Wear rate.

To elucidate the wear mechanisms of high-Nb TiAl alloys, white light interferometry was employed to characterize the wear track topography, and wear rates were calculated. As shown in Figure 5d and Figure 6, the wear track widths for VED-1 to VED-5 were 1399.02 μm, 971.71 μm, 977.56 μm, 1190.30 μm, and 1592.19 μm, respectively, with corresponding depths of 39.19 μm, 21.83 μm, 25.30 μm, 26.75 μm, and 55.09 μm. These dimensional measurements conclusively demonstrated that the VED-2 specimen possesses the highest wear resistance among the cohorts, evidenced by its minimal material wear rate. Consistent with these morphologies, the wear rate followed a similar evolution trend, with VED-2 achieving the lowest calculated wear rate value of 2.9164 × 10^−6^ mm^3^/N·m. However, VED-5 has the highest hardness but the highest wear resistance. This can be attributed to the transformation of the wear mechanism driven by structural integrity. In the defect-free VED-2, the friction stress is evenly distributed, allowing for the formation of a stable, adherent mechanical mixed layer composed of Ti/Al oxides. This “glaze layer” acts as a protective barrier, maintaining a slight oxidative wear. In contrast, in VED-5, the internal large pores and internal microcracks act as stress concentrators. Under cyclic Hertzian contact stress, these critical defects exceed the fracture toughness threshold of the surrounding brittle B2 matrix, triggering rapid underground crack propagation and large-scale delamination, whereas the fine spherical pores in VED-2 safely distribute the load. The detached debris, due to the characteristics of the B2 phase, is extremely hard and brittle and gets trapped at the sliding interface, exacerbating the material removal through severe tri-body abrasive wear mechanism.

**Figure 7:**
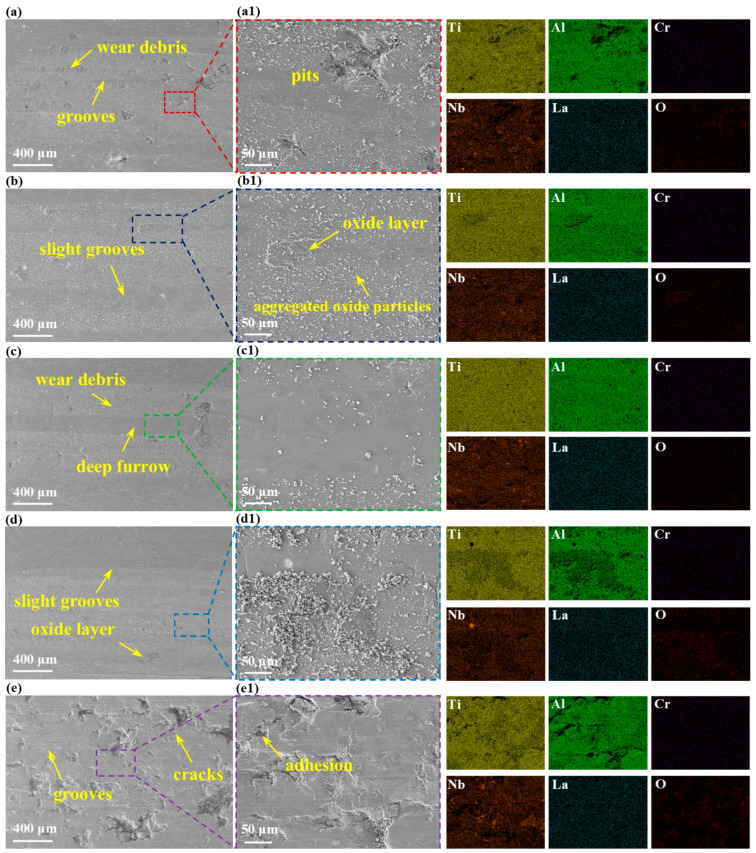
Wear morphology and EDS analysis of high-Nb TiAl specimens: (**a**) VED-1; (**a1**) Enlarged view of (**a**); (**b**) VED-2; (**b1**) Enlarged view of (**b**); (**c**) VED-3; (**c1**) En-larged view of (**c**); (**d**) VED-4; (**d1**) Enlarged view of (**d**); (**e**) VED-5; (**e1**) Enlarged view of (**e**).

Figure 7 presents the SEM images and EDS mapping of the worn morphologies of different specimens. Generally, tribological failure was governed by the synergy of adhesive, oxidative, abrasive, and fatigue wear [32]. Due to the high velocity and high-pressure sliding, frictional heating induced an instantaneous high temperature at the contact interface, accelerating the oxidation of surface constituents. Subsequent EDS analysis identified the resulting tribo-film and particulate wear debris as predominantly Ti- and Al-based oxides. However, the delamination typically triggered a transition from two-body contact to severe three-body abrasion. Furthermore, according to the Hertzian contact theory [33], the subsurface region located in the maximum shear stress induced the nucleation of microcracks. When the accumulated stress value exceeded the fracture toughness of the surface layer, these cracks propagated outward, resulting in material spalling. These detached, hard oxide fragments were comminuted into fine debris, which was entrapped within the sliding interface and exacerbates volume loss by three-body abrasion. Specific observations revealed that the VED-2 specimen exhibited a relatively smooth worn surface characterized only by shallow plowing grooves parallel to the sliding direction, as shown in Figure 7b. This superior performance was attributed to the high structural integrity and low defect density of the specimen. However, the VED-5 specimen displayed a fragmented protective layer due to the poor forming quality. The prevalence of deep gouges and scattered debris confirmed the dominance of abrasive and oxidative wear.

**Figure 8:**
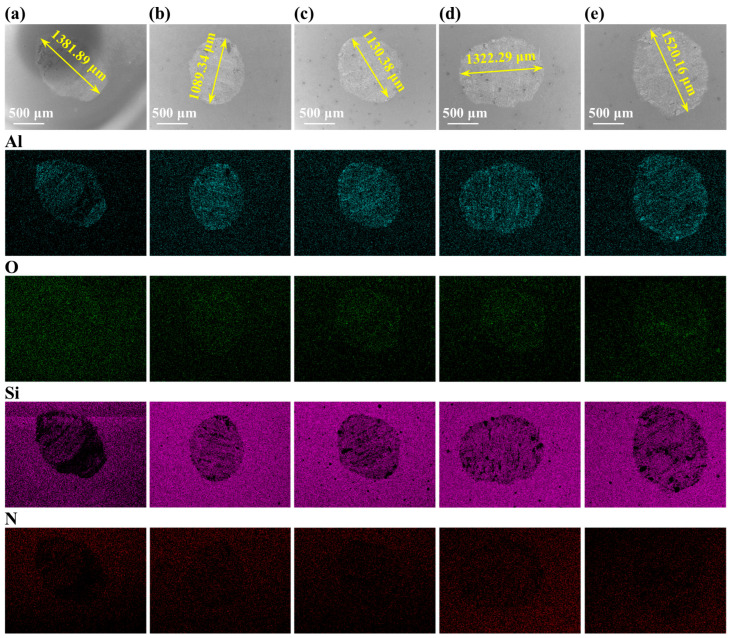
Worn surfaces of the counter balls corresponding to specimens: (**a**) VED-1; (**b**) VED-2; (**c**) VED-3; (**d**) VED-4; (**e**) VED-5.

To corroborate the wear mechanisms proposed above, the surface morphologies of the counterpart grinding balls are characterized, as displayed in Figure 8. There was a distinct correlation between the wear dimension on the Si_3_N_4_ balls and the wear rates of high-Nb TiAl specimens. The counterparts corresponding to the VED-1 and VED-5 specimens exhibited extensive material loss and rough surfaces, which confirmed the presence of abrasive particles. Conversely, the Si_3_N_4_ balls corresponding to VED-2 showed the smallest wear diameters and a smooth surface. The slight grooves indicated a stable friction interface and a coherent oxide film that protected the substrate specimen. However, chemical composition analysis revealed the presence of Al, O, Si, and N, while Ti was notably absent. This element distribution confirmed that Al_2_O_3_ was predominantly generated on the specimen surface and subsequently transferred to the counterpart.

### 3.4. Corrosion Resistance

**Figure 9:**
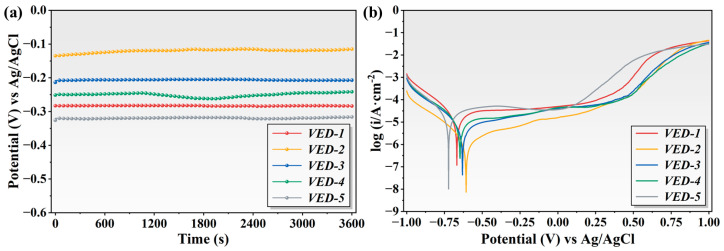
(**a**) Open circuit potential measurements, (**b**) Potential polarization curves of LPBF-fabricated high-Nb-TiAl alloys.

Figure 9a presents the open circuit potential profiles for the high-Nb-TiAl alloys. The potentials of all specimens gradually shifted in a positive direction, eventually reaching a steady state, indicating the spontaneous formation of a passive film on the surfaces during immersion. The electrochemical behavior and corrosion resistance of high-Nb TiAl alloys, fabricated at varying laser volume energy densities, were investigated in 3.5 wt.% NaCl solution using potentiodynamic polarization. As illustrated in Figure 9b, the polarization curves delineate the surface dynamic response during the potential scan from the cathodic to the anodic region. The morphologies of the polarization curves for different specimens are comparable, suggesting similar corrosion mechanisms and passivation behavior. In the cathodic region, the current density gradually decreased as the potential shifted positively, accompanied by extensive dissolution redox reactions. As the corrosion potential approached the anodic region, the polarization behavior of the specimens diverged significantly. The VED-2 specimen exhibited a gradual increase in current density with increasing anodic potential, with the widest passive region. This suggests the formation of a stable, dense passivation film that effectively inhibited chloride ion attack and metal dissolution. Conversely, the VED-5 and VED-1 specimens showed a steeper rise in current density beyond the corrosion potential, indicating inferior stability and protective performance of their passivation film that are susceptible in the passive region. At potentials exceeding 0.75 V, the polarization curves converged as the specimen surfaces entered the inactive region.

The quantitative electrochemical parameters, including corrosion potential (*E*_corr_), corrosion current density (*i*_corr_), and the cathodic and anodic Tafel slopes (*β_cat_*, *β_ano_*), were determined from polarization curves via the Tafel extrapolation method and are summarized in Table 2. Corrosion potential serves as a significant indicator of thermodynamic susceptibility to corrosion. Generally, the higher potential implies superior corrosion resistance [34]. With increasing energy density, *E*_corr_ initially rose before declining, following the order: VED-2 > VED-3 > VED-4 > VED-1 > VED-5. Specimen VED-2 exhibited the highest *E*_corr_, suggesting maximal thermodynamic stability within the corrosive environment. Conversely, VED-5 exhibited a substantially lower *E*_corr_ and a higher corrosion current density, indicating inferior corrosion resistance. Since *i*_corr_ is directly proportional to the corrosion rate, lower values correspond to enhanced kinetic resistance [35]. The specimens were ranked in ascending order of *i*_corr_: VED-5 > VED-1 > VED-4 > VED-3 > VED-2. Notably, the *i*_corr_ of VED-5 was approximately 14.5 times that of VED-2, suggesting rapid dissolution of the passive film and significantly diminished corrosion resistance.

Analysis of the cathodic and anodic Tafel slopes further elucidated the corrosion mechanism. As detailed in Table 2, the reactions follow distinct kinetic pathways, with the cathodic rate being significantly more sensitive to potential changes than the anodic rate. The elevated cathodic Tafel slope suggests sluggish kinetics, in which current density responds slowly to potential shifts, indicating the reduction of dissolved oxygen. This observation aligns with the well-established consensus that oxygen diffusion primarily controls the corrosion of titanium alloys. Conversely, the lower anodic Tafel slope indicates the inherent corrosion resistance of the alloys [36]. Titanium and its alloys readily form a dense, stable passivation film in oxygenated environments. This passive layer effectively inhibits anodic dissolution, maintaining low current densities over a wide potential range. Consequently, the anodic reaction becomes less sensitive to potential variations, resulting in the observed lower Tafel slope.

**Figure 10:**
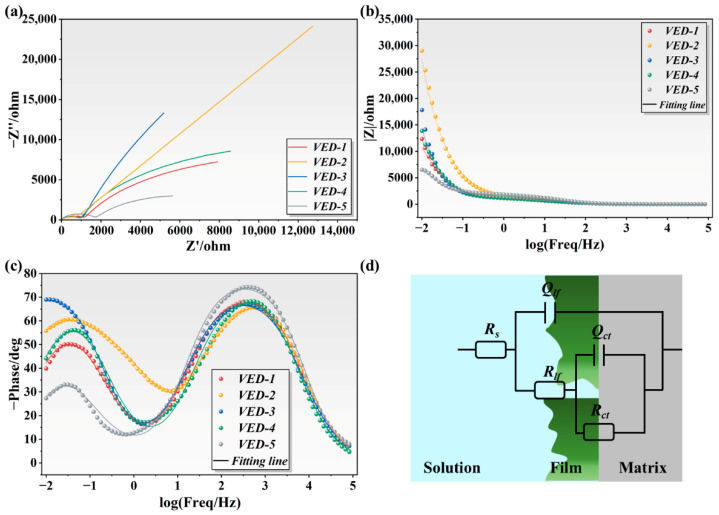
The EIS results of high-Nb TiAl alloys in 3.5 wt.% NaCl solution: (**a**) Nyquist plots; (**b**,**c**) Bode plots; (**d**) Equivalent circuit.

EIS measurements were performed to evaluate the compactness and stability of the passive film formed on the high-Nb TiAl alloys in 3.5 wt.% NaCl solution. The resulting Nyquist and Bode plots, along with the fitted equivalent electrical circuits, are presented in Figure 10, with the data simulation facilitated by ZSimpwin 3.6 software. The Nyquist plots exhibit a characteristic dual-time-constant behavior, manifesting as a capacitive loop at high frequencies and a linear trajectory at lower frequencies. The high-frequency response is attributed to the dielectric properties of the surface film, specifically associating the arc morphology with the outer porous layer. Notably, the relatively small radius of these capacitive loops suggests that this outer porous structure offers only a marginal physical barrier against electrolyte permeation. Conversely, in the low-frequency domain, the linear tail corresponds to the electrochemical responses of the compact inner barrier layer. This inner passive region was the primary obstacle to ion diffusion, and its impedance magnitude was the critical determinant of the corrosion resistance.

The Bode plots, which include the impedance modulus |Z| and the phase angle spectra, offer complementary insights into the interfacial kinetics. In the low-frequency regime, an elevated |Z| indicates a high charge-transfer resistance and sluggish electrode kinetics, which are hallmarks of superior corrosion resistance [37]. Consistent with the Nyquist analysis, the Bode magnitude data demonstrate that the VED-2 specimen maintains the highest |Z| values across the investigated frequency spectrum of 10^−2^–10^5^ Hz. This spectral feature signifies the formation of a robust passive architecture that is resilient against attack by aggressive ions. Conversely, the depressed |Z| values observed for VED-5 reflect its comparatively compromised protective performance. Furthermore, the phase angle maximum serves as a critical diagnostic criterion for film integrity. As depicted in Figure 10c, a shift towards more negative phase angles implies a compact dielectric layer. The comprehensive EIS analysis substantiates that VED-2 possesses superior corrosion resistance among the high-Nb TiAl alloys.

The quantitative parameters derived from the EIS measurements are summarized in Table 3, providing critical insights into the corrosion mechanism and the evolution of the phase film. The goodness of fit for the proposed equivalent electrical circuits was assessed using the χ^2^ statistic, which confirmed that the simulation errors are within an acceptable statistical range. Fitted parameters *R_s_*, *R_f_*, and *R_ct_* denote the solution resistance, film resistance, and charge transfer resistance, respectively. Notably, *R_ct_* is magnitudes larger than *R_f_*, suggesting that the electrochemical kinetics are primarily governed by charge transfer at the interface rather than by ion migration through the film. Specifically, the VED-2 specimen exhibits an *R_ct_* of approximately 2.73 × 10^4^ Ω·cm^2^, a substantial increase compared with the VED-5 specimen. This elevated resistance implies a significant kinetic barrier to charge transfer, effectively inhibiting the anodic dissolution reaction. Furthermore, the dispersion coefficient for the VED-2 specimen is lower than that of its counterpart, indicating a thicker passive film on its surface, which provided more effective protection of the matrix from corrosion.

**Figure 11:**
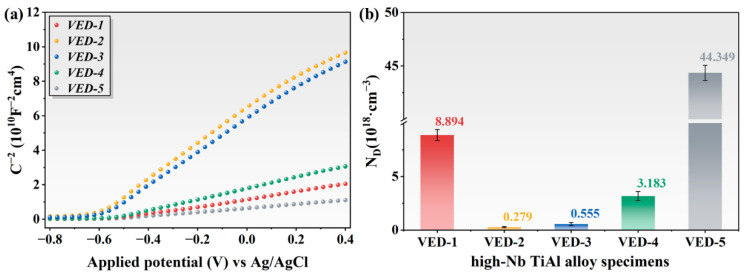
Mott-Schottky curves and carrier density values of the passive film of high-Nb TiAl alloy in the 3.5 wt.% NaCl solution: (**a**) M-S curves, (**b**) carrier density values.

The Mott-Schottky (MS) curves and carrier density value of the passive film of high-Nb TiAl alloy in the 3.5 wt.% NaCl solution. As depicted in Figure 11a, the linear region with a positive slope across the potential interval −0.8 to 0.4 V confirmed that the passive film behavior was characteristic of an n-type semiconductor. The electronic behavior suggests that charge transfer at the film-electrolyte interfaces is modulated by donor species, predominantly arising from point defects such as oxygen vacancies and cation interstitials. Quantitatively, the donor carrier density (*N_D_*) is calculated based on the MS relationship defined as [38]:

(5) $$\frac{1}{C^{2}}=\frac{2}{\varepsilon \cdot \varepsilon_{0}\cdot e\cdot N_{D}}\left[E-E_{fb}-\frac{kT}{e}\right]$$

where *C* is the capacitance of the space charge layer, *ε* is the relative dielectric constant of the film (*ε* = 15.6), *ε*_0_ denotes the vacuum dielectric constant (*ε*_0_ = 8.854 × 10^−14^ F/cm), *e* is the elementary charge (*e* = 1.6 × 10^−19^ C), *N_D_* is the donor concentration, *E* is the applied potential, *E_fb_* is the flat band potential, *k* is the Boltzmann constant (*k* = 1.38 × 10^−23^ J/K), and *T* is the absolute temperature. The *N_D_* values were calculated through the slope of the linear region in the *C*^−2^ versus *E* plots. During the corrosion process, oxygen vacancies significantly promote electron transfer in electrochemical reactions, thereby increasing susceptibility to corrosion [39]. The *N_D_* calculation values were summarized in Figure 11b. The VED-2 has the lowest carrier density value of 0.279 × 10^18^ cm^−3^ among the investigated specimens, indicating a more compact and stable passive film.

**Figure 12:**
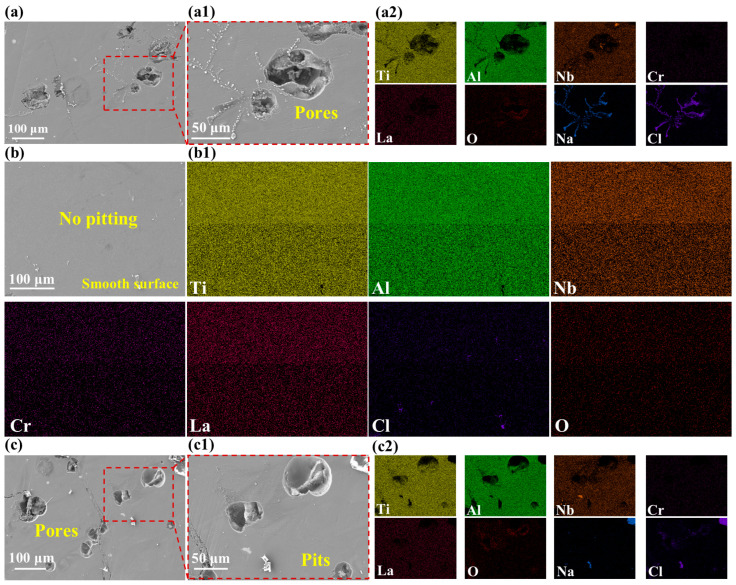
Corrosion morphology and EDS mapping of high-Nb TiAl alloy: (**a**) VED-1; (**a1**) Enlarged view of (**a**); (**a2**) EDS scanning results shown in (**a1**); (**b**) VED-2; (**b1**) EDS scanning results shown in (**b**); (**c**) VED-5; (**c1**) Enlarged view of (**c**); (**c2**) EDS scanning results shown in (**c1**).

To visually corroborate the electrochemical reaction mechanisms, the surface morphologies of the high-Nb TiAl alloys following potentiodynamic polarization measurements, along with the corresponding EDS elemental mappings, are presented in Figure 12. Distinct variations in corrosion resistance are evident across the specimens. The surfaces of VED-1 and VED-5 exhibited severe localized degradation, characterized by deep, cavernous pits and extensive matrix exfoliation, signifying a catastrophic breakdown of the protective passive film. While dissolved oxygen initially induces the formation of a passive oxide layer on the high-Nb TiAl alloy surface, the preferential adsorption of aggressive Cl^−^ ions at surface defects inevitably triggers local film rupture as corrosion progresses. Conversely, the VED-2 specimen retained a remarkably intact and homogeneous topography, suggesting that the passive film formed on this specimen was highly compact and stable, thereby effectively shielding the substrate from electrolyte attack. This failure mechanism is further elucidated by the EDS analysis. Within the corrosion cavities of VED-1 and VED-5, a pronounced depletion of Ti and Al elements was observed, accompanied by a significant enrichment of Cl and Na. This elemental segregation provides direct evidence of pitting corrosion. Following the film breakdown, the hydrolysis of dissolved metal cations within the occluded pits reduces the local hydrogen potential. To maintain electroneutrality, chloride ions migrate from the bulk solution into the pits, creating a highly acidic, chloride-rich environment that accelerates anodic dissolution. In contrast, the uniform elemental distribution observed on VED-2 confirms that its optimized microstructure mitigates the ingress of aggressive ions, effectively suppressing the transition from metastable nucleation to stable pit growth. Moreover, electrochemical stability is intrinsically governed by the spatial chemical homogeneity within the solidified melt pool. EDS analysis identified severe Nb segregation in the VED-5 specimen. Electrochemically, such compositional gradients established local micro-galvanic cells, wherein the noble Nb-enriched regions function as cathodes coupled with the active Ti/Al anodic matrix. Because Nb possesses a significantly more positive standard equilibrium potential than the more active Ti and Al constituents, it invariably acts as an efficient local micro-cathode. This substantial thermodynamic potential gradient across the segregated domains provides the electromotive force necessary to accelerate the preferential anodic dissolution of the adjacent, less noble Ti/Al matrix. However, the enhanced Marangoni convection in VED-2 facilitated solute homogenization, thereby attenuating the thermodynamic driving force for localized corrosion.

Fundamentally, corrosion resistance is intrinsically governed by the microstructural integrity of the high-Nb TiAl surface. For the defect-free specimen, the high surface densification promotes the development of a coherent and uniform passive film, which acts as a formidable physical barrier retarding the adsorption and ingress of aggressive Cl^−^ ions [40]. Conversely, porosity not only extends the effective electrolyte-electrode interface but also provides preferential nucleation sites for the pitting. Unlike the fine and isolated pores observed in VED-2, which can be effectively sealed by the passive oxide layer, these large macro-defects disrupt the continuity of the passive film, hinder uniform electrolyte distribution, and provide preferential unpassivated pathways for Cl^−^ ingress and local acidification, thereby compromising local film protectiveness. Notably, the crack-pore specimen exhibits the most severe corrosion. This deterioration is attributed to the strong galvanic driving force created by the local oxygen-rich anode. This electrochemical effect accelerates anodic dissolution and electrolyte diffusion, resulting in catastrophic localized corrosion within the crevice regions.

**Figure 13:**
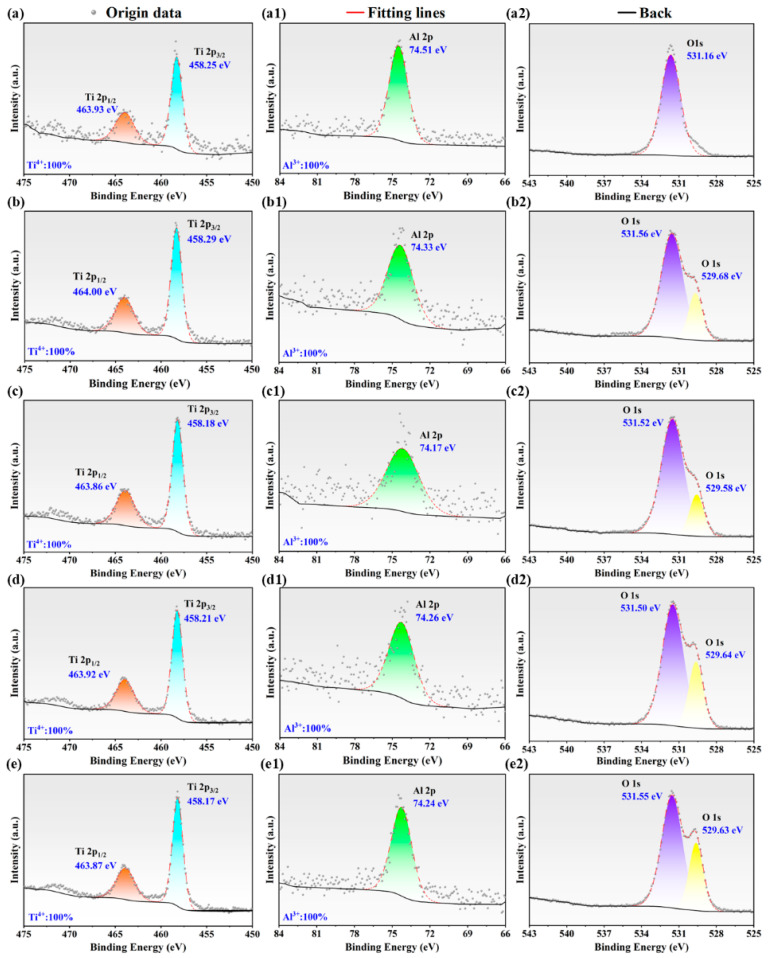
XPS spectra of high-Nb TiAl alloys: (**a**–**a2**) Spectra to Ti, Al, and O in VED-1; (**b**–**b2**) Spectra to Ti, Al, and O in VED-2; (**c**–**c2**) Spectra to Ti, Al, and O in VED-3; (**d**–**d2**) Spectra to Ti, Al, and O in VED-4; (**e**–**e2**) Spectra to Ti, Al, and O in VED-5.

X-ray photoelectron spectroscopy (XPS) was employed to characterize the chemical composition of the passive films formed on the high-Nb TiAl alloys. As shown in Figure 13, the survey spectra across varying microstructures exhibited analogous elemental profiles, predominantly comprising Ti 2p, O 1s, and Al 2p species, with the adventitious C 1s peak ascribed to environmental exposure. In the high-resolution Ti 2p spectra, the Ti 2p_3/2_ binding energy for all specimens clustered between 458.1 eV and 458.3 eV, a range characteristic of the TiO_2_ phase. Distinctly, VED-2 exhibited the largest binding energy shift, which demonstrated superior corrosion resistance. This suggested a passive film dominated by the fully oxidized Ti^4+^ state, implying a minimized fraction of sub-stoichiometric low-valent cations, such as Ti^3+^ and Ti^2+^. Their low-valent titanium species function as proxies for oxygen vacancies within the oxide matrix. Conversely, VED-5 displayed the lowest binding energy, indicating a significant proportion of sub-oxides. This sub-stoichiometry correlates with an increased defect and compromised film stability, corroborating the elevated carrier density observed in the MS analysis. These findings were further substantiated by the deconvolution of O 1s spectra, which resolved into lattice oxygen and adsorbed oxygen-hydroxyl groups. For the VED-2 specimen, the pronounced lattice oxygen peak at 529.68 eV indicated the formation of a continuous, dense metal-oxide framework that served as a robust physical barrier against electrolyte ingress. Beyond TiO2, aluminum oxides play a pivotal role in enhancing passivation behavior. The Al 2p spectra showed peak centers ranging from 74.1 eV to 74.6 eV, confirming the complete oxidation of surface aluminum to the Al^3+^ state, which corresponds to a thermodynamically stable and dense Al_2_O_3_ phase. Therefore, the incorporation of Al_2_O_3_ into the TiO_2_ matrix generated a synergistic effect that likely mitigates structural defects, which endowed VED-2 with the highest impedance modulus.

Post-polarization surface chemistry was characterized using XPS analysis to elucidate compositional variations across specimens, as displayed in Table 4. Given the superior thermodynamic stability and dielectric nature of titanium oxides in aggressive environments, the surface Ti atomic concentration was a critical determinant for the barrier efficacy of the passive film [41]. Notably, VED-2 and VED-3 retained the highest Ti atomic fractions, reaching 11.10% and 11.12%, respectively. Such enrichment suggested that a compact Ti-rich passivation layer remained stable under electrochemical stress, thereby mitigating the ingress of corrosive species and corroborating the superior corrosion resistance observed in these specimens. Conversely, VED-5 underwent marked surface reconstruction, evidenced by a substantial depletion of Ti alongside a concurrent enrichment of Al. The resulting low Ti/Al ratio implied the dissolution or structural compromise of the stable TiO_2_ barrier, leading to a surface dominated by thermodynamically less stable aluminum oxides. Consequently, the diminished protective capacity accounts for the inferior corrosion performance of the VED-5. Meanwhile, VED-1 and VED-4 exhibited intermediate elemental profiles, indicating moderate passivation kinetics falling between the aforementioned extremes. Therefore, in this study, the high-Nb TiAl alloy passivation film was mainly composed of TiO_2_, which was formed by the electrochemical process [42]:

(6) $$2Ti+3H_{2}O\to Ti_{2}O_{3}+6H^{+}+6e^{-}$$

(7) $$Ti_{2}O_{3}+3H_{2}O\to 2TiO{\left(OH\right)}_{2}+2H^{+}+2e^{-}$$

(8) $$Ti+3H_{2}O\to TiO{\left(OH\right)}_{2}+2H^{+}+2e^{-}$$

(9) $$TiO+{\left(OH\right)}_{2}\to TiO_{2}+H_{2}O$$

## 4. Conclusions

This study systematically investigated the influence of VED on the microstructure, wear, and corrosion resistance of high-Nb TiAl alloys fabricated by LPBF. The key conclusions are summarized as follows:

(1) During the LPBF process, optimizing the VED facilitated significant densification, elevating the relative density of the specimens to 97.55%. However, increasing or decreasing the energy input exacerbated the proliferation of metallurgical defects, particularly porosity and micro-cracks.
(2) The microstructure of the high-Nb TiAl alloy predominantly comprises γ, α2, and β/B2 phases. This phase constitution was fundamentally dictated by the non-equilibrium rapid solidification inherent to LPBF, combined with the strong β-stabilizer element Nb, which retards the diffusional β → α transformation.
(3) Specifically, at a VED of 81.48 J/mm^3^, the as-built specimen attained a hardness of 422 HV_0.5_ due to the high proportion of α2 and B2 phase. Specimen VED-2 demonstrated superior tribological performance with the narrowest wear track dimension and lowest wear rate. With the increased VED, the wear mechanism shifted from severe delamination and three-body abrasive wear to mild abrasive wear accompanied by plowing.
(4) The corrosion behavior was intrinsically linked to the continuity of the passive films. The VED-2 specimen exhibited the lowest corrosion current density of 1.421 × 10^−6^ A/cm^2^. This enhanced resistance is attributed to a coherent passive oxide film that effectively impeded the ingress of corrosive ions, thereby mitigating the corrosion reaction. Conversely, in specimens with suboptimal VED, pores and cracks served as active sites for electrolyte stagnation and Cl^−^ enrichment, thereby accelerating the corrosion of specimens.

## Figures and Tables

**Table 1:** LPBF manufacturing process parameters.

Sample	*P* (W)	*v* (mm/s)	VED (J/mm^3^)
S_70-900_	70	900	51.85
S_90-900_	90	900	66.67
S_110-900_	110	900	81.48
S_130-900_	130	900	96.30
S_150-900_	150	900	111.11
S_110-600_	110	600	122.22
S_110-700_	110	700	104.76
S_110-800_	110	800	91.67
S_110-1000_	110	1000	73.33
S_110-1100_	110	1100	66.67

**Table 2:** Electrochemical parameter results of the polarization curve analysis performed on specimens.

Specimens	*E*_corr_ (V)	*i*_corr_ (A·cm^−2^)	−*β_cat_* (V/dec)	*β_ano_* (V/dec)
VED-1	−0.668 ± 0.018	1.710 ± 0.017 × 10^−5^	6.927 ± 0.033	3.077 ± 0.024
VED-2	−0.607 ± 0.013	1.421 ± 0.025 × 10^−6^	6.967 ± 0.029	3.206 ± 0.016
VED-3	−0.631 ± 0.021	6.410 ± 0.016 × 10^−6^	7.027 ± 0.037	2.809 ± 0.022
VED-4	−0.647 ± 0.011	1.200 ± 0.027 × 10^−5^	6.340 ± 0.018	2.183 ± 0.017
VED-5	−0.723 ± 0.017	2.060 ± 0.023 × 10^−5^	6.631 ± 0.025	3.144 ± 0.031

**Table 3:** Fitting results from EIS data.

Parameter	VED-1	VED-2	VED-3	VED-4	VED-5
*R_s_* (Ω·cm^2^)	6.60 (±0.12)	7.06 (±0.77)	6.50 (±0.86)	8.23 (±1.21)	6.49 (±1.02)
*Q_f_*-*Y_0_* (F·cm^−2^ × 10^−5^)	1.81 (±0.66)	1.71 (±0.53)	2.19 (±0.41)	1.43 (±0.64)	1.03 (±0.47)
*Q*-n1	0.83 (±0.09)	0.84 (±0.04)	0.83 (±0.06)	0.85 (±0.02)	0.89 (±0.06)
*R_f_* (Ω·cm^2^)	1208 (±6.12)	725.9 (±4.25)	1106 (±5.36)	990.3 (±5.04)	1719 (±6.83)
*Q_dl_*-*Y_0_* (F·cm^−2^ × 10^−4^)	7.14 (±0.77)	2.47 (±0.45)	7.46 (±0.78)	6.86 (±0.57)	10.72 (±0.85)
*Q*-n2	0.81 (±0.39)	0.75 (±0.34)	0.78 (±0.24)	0.78 (±0.37)	0.84 (±0.22)
*Rct* (Ω·cm^2^ × 10^4^)	1.07 (±0.19)	2.73 (±0.53)	1.43 (±0.24)	1.21 (±0.46)	0.64 (±0.37)
χ^2^ (×10^−3^)	0.30	1.12	1.84	0.98	2.07

**Table 4:** Semi-quantitative analysis of elemental atomic concentrations from XPS spectra.

Atomic Fraction (%)	VED-1	VED-2	VED-3	VED-4	VED-5
Ti	7.25	11.10	11.12	10.21	3.60
Al	3.91	4.74	8.39	6.31	10.01
O	88.84	84.16	80.49	83.48	86.39

## Data Availability

The original contributions presented in this study are included in the article. Further inquiries can be directed to the corresponding authors.
